# Literature Mining and Mechanistic Graphical Modelling to Improve mRNA Vaccine Platforms

**DOI:** 10.3389/fimmu.2021.738388

**Published:** 2021-09-07

**Authors:** Lorena Leonardelli, Giuseppe Lofano, Gianluca Selvaggio, Silvia Parolo, Stefano Giampiccolo, Danilo Tomasoni, Enrico Domenici, Corrado Priami, Haifeng Song, Duccio Medini, Luca Marchetti, Emilio Siena

**Affiliations:** ^1^Fondazione The Microsoft Research – University of Trento Centre for Computational and Systems Biology (COSBI), Rovereto, Italy; ^2^Preclinical, GSK, Rockville, MD, United States; ^3^Department of Cellular, Computational and Integrative Biology (CIBIO), University of Trento, Povo, Italy; ^4^Department of Computer Science, University of Pisa, Pisa, Italy; ^5^Toscana Life Sciences Foundation, Siena, Italy; ^6^Data Science and Computational Vaccinology, GSK, Siena, Italy

**Keywords:** mRNA vaccines, natural language processing, graphical modeling, scientific literature mining, mechanisms of action

## Abstract

RNA vaccines represent a milestone in the history of vaccinology. They provide several advantages over more traditional approaches to vaccine development, showing strong immunogenicity and an overall favorable safety profile. While preclinical testing has provided some key insights on how RNA vaccines interact with the innate immune system, their mechanism of action appears to be fragmented amid the literature, making it difficult to formulate new hypotheses to be tested in clinical settings and ultimately improve this technology platform. Here, we propose a systems biology approach, based on the combination of literature mining and mechanistic graphical modeling, to consolidate existing knowledge around mRNA vaccines mode of action and enhance the translatability of preclinical hypotheses into clinical evidence. A Natural Language Processing (NLP) pipeline for automated knowledge extraction retrieved key biological evidences that were joined into an interactive mechanistic graphical model representing the chain of immune events induced by mRNA vaccines administration. The achieved mechanistic graphical model will help the design of future experiments, foster the generation of new hypotheses and set the basis for the development of mathematical models capable of simulating and predicting the immune response to mRNA vaccines.

## Introduction

**Figure 1:**
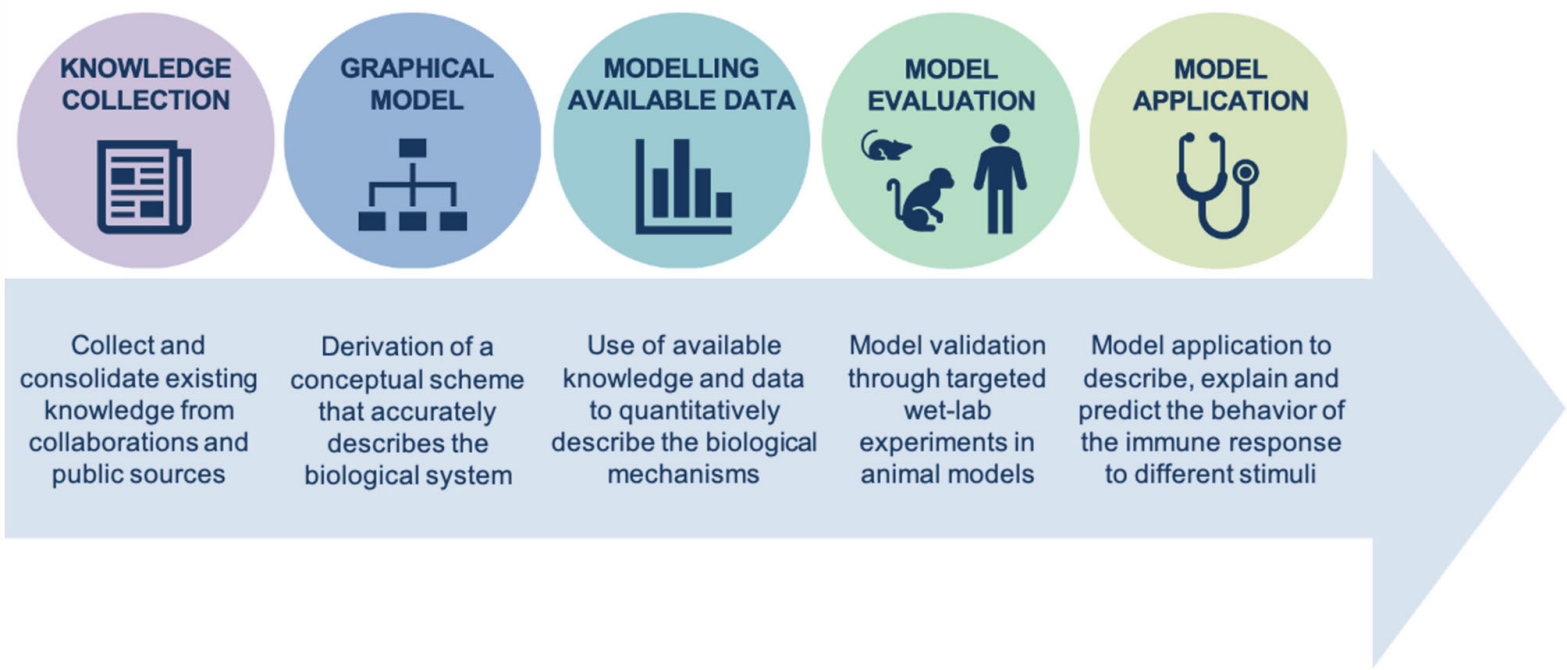
*In silico* modeling of the immune system. Integrative systems biology approach to properly describe, explain, and predict the behavior of any biological mechanism, which in our specific case study is the immune response to mRNA-based vaccines.

Since December 2019 SARS-CoV-2 virus has spread across the globe, becoming a pandemic threat and claiming millions of lives. The contagiousness combined with the mortality rate triggered unprecedented efforts to quickly design and develop a vaccine. The first two vaccines that received emergency use authorization by EMA and FDA to prevent COVID-19 disease in humans are based on messenger RNA (mRNA), a relatively new vaccine platform with several advantages over more traditional approaches for vaccine design (1–7). Because of their unique features in terms of manufacturability, mechanism of action and ability to induce potent immune responses, mRNA vaccines represent an important advancement in the history of vaccinology to defeat infectious diseases. mRNA vaccines are based on the concept that, starting from the amino acid sequence of the antigen of interest, it is possible to design a related mRNA sequence that is employed by the cells of the body as template to express the antigen *in situ*. mRNA vaccines are known to stimulate both arms of the humoral and cellular immunity (7), however their mechanism of action is still partially understood. In this work, we applied a systems biology approach (**Figure 1**) to dissect and elucidate: the delivery of mRNA vaccines, the antigen expression and the resulting vaccine-specific immune responses.

The strategy depicted in **Figure 1** applies to several biological processes and sets the basis for building mathematical models starting from already established knowledge, mainly stored within the scientific literature. The ability for an investigator to process the vast amount of publications through automated literature mining and natural language processing (NLP) algorithms is instrumental to efficiently collect relevant information and define a comprehensive picture. Within each publication, the heterogeneity of data sources (e.g. *in vitro* evidence, animal models, clinical trials, etc.) requires an additional effort to integrate the retrieved information into an interactive platform that can be queried by the users. Databases are usually the preferred solution to store parameters and other numerical information, but as per RNA vaccines some processes may particularly benefit from a mechanistic graphical model that would help identify and fill in the knowledge gaps. Indeed, literature derived data could be complemented with proprietary knowledge and used to draft a mathematical model of the underlying biology. Accordingly, progressive and iterative refinement of the mechanistic process through targeted wet-lab experiments in animal models contributes to the model evaluation. The finalized model is eventually applied to specific scenarios of interest to predict, investigate and support drug development.

**Figure 2:**
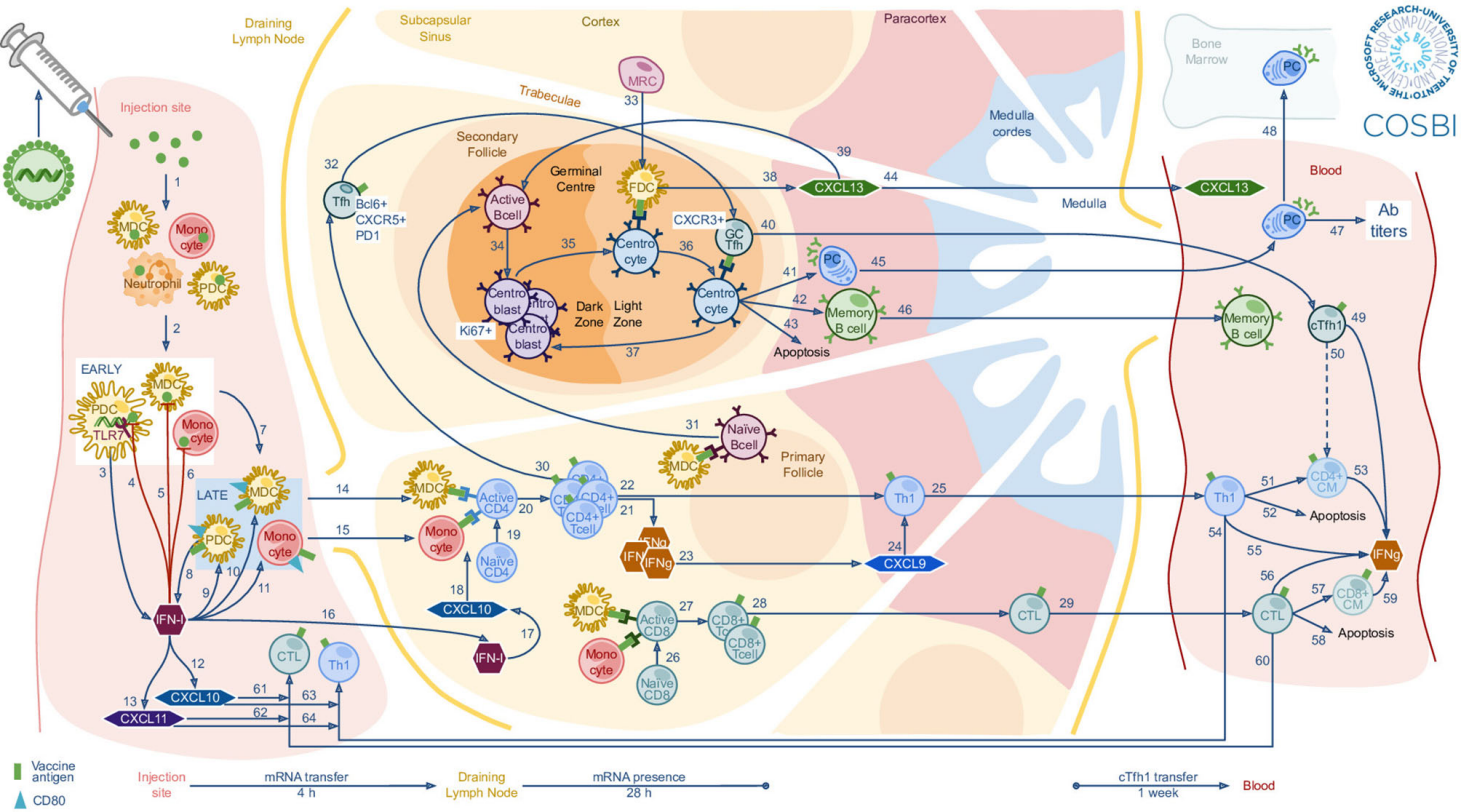
Mechanistic graphical model of the mRNA-based vaccine biological mechanism of action. Lipid nanoparticles encapsulating mRNA molecules are intradermal or intramuscular injected triggering immune signals that reach the draining lymph node, where other immune reactions generate the specific antibodies, eventually transferred into the circulatory system. This figure is proposed in a dynamic view at the link https://www.cosbi.eu/fx/9839203, where each connection (blue arrows) is documented by literature references as well as the involved cells measured in mouse and NHP experiments.

Although mRNA vaccines have been the focus of several studies in the last decade, they are usually employed with different components (encoded antigen, delivery system, mRNA architecture) and investigations focused on different arms of the immune response or different biological sites. Consequently, the generated knowledge tends to be rather specific to each individual platform, not always generalizable and sometimes even fragmented. This is what motivated our effort to collect and consolidate all publicly available scientific evidences, related to mRNA vaccine mode of action, into an interactive mechanistic graphical model (**Figure 2**, link to https://www.cosbi.eu/fx/9839203 dynamic figure) tracing the stages of the immune response to mRNA vaccines, from the innate immune activation at the site of injection up to the adaptive response, measurable in the peripheral blood system several days after vaccination. The proposed mechanistic graphical model facilitates the interpretation of what has been discovered so far and fosters interactions among investigators with very different backgrounds, such as immunologists and data scientists.

## Literature Mining and Information Processing

Automated knowledge extraction from text is the very definition of text-mining, a process becoming more and more a necessity in researching the everyday growing amount of available body of text (8). Text mining does not just find the documents reporting the searched information, but systematically and efficiently examines those contents, a process that performed manually would be unfeasible, unrealistic and error prone. Several tools have been already developed for extracting biomedical information (9, 10), based on sophisticated NLP algorithms to read and analyze the huge number of scientific publications. Over time those methods have been largely improved, becoming suitable for annotating large experimental datasets and merging data from several studies across the world, which may lead to the discovery of global trends within the existing literature (11, 12).

In this part of the study we investigated the biological perturbations, at the level of immune cells and immunoactive peptides (cytokines/chemokines and cell-surface markers), induced by mRNA vaccines through an NLP-guided literature mining process (10). The initial search for mRNA vaccines-associated literature identified 361 scientific articles in PubMed, 245 of which were automatically annotated as providing experimental evidences generated in animal models. Given the relevance of non-human primates (NHP) as a model for human immunology studies, the pipeline selected 17 NHP-related papers, among which 6 were defined as eligible sources of information and manually confirmed (**Figure 2**). The 6 retrieved scientific publications have been extremely useful to understand the available information about mRNA vaccine mode of action in NHP and one described the SAM platform. To be as inclusive as possible, mice-derived data and information were also taken into consideration. These consisted in a set of 9 self-amplifying mRNA vaccines studies, 5 of which provided eligible quantitative information (**Table 1**). Manually curating the 361 papers of interest since the beginning would have been highly time consuming, while the properly instructed NLP pipeline was able to provide the answers to our questions in a matter of few hours, leaving to the scientists a manageable amount of information to explore.

**Table 1:** List of relevant scientific literature studying mRNA-based vaccines in mouse and NHP animal models.

PMID	Title	Journal	Year	First Author	Animal model	Vaccine features
30936432 (13)	Visualization of early events in mRNA vaccine delivery in non-human primates *via* PET-CT and near-infrared imaging.	Nat Biomed Eng	2019	Lindsay KE	NHP	Yellow fever (YF) prME mRNA vaccine complexed with lipid derivatives
29739835 (4)	Nucleoside-modified mRNA vaccines induce potent T follicular helper and germinal center B cell responses.	J. Exp. Med.	2018	Pardi N	mice, NHP	3 vaccines: mRNA-LNPs encoding HIV-1 envelope (Env), ZIKV prM-E, and influenza virus hemagglutinin (HA)
28958578 (14)	Efficient Targeting and Activation of Antigen-Presenting Cells *In Vivo* after Modified mRNA Vaccine Administration in Rhesus Macaques.	Mol. Ther.	2017	Liang F	NHP	LNP-mRNA encoding hemagglutinin (HA) of H10N8 influenza A virus (H10)
29181005 (15)	Induction of Robust B Cell Responses after Influenza mRNA Vaccination Is Accompanied by Circulating Hemagglutinin-Specific ICOS+ PD-1+ CXCR3+ T Follicular Helper Cells.	Front Immunol	2017	Lindgren G	NHP	LNP-mRNA encoding hemagglutinin (HA) of H10N8 influenza A virus (H10)
25234719 (16)	Potent immune responses in rhesus macaques induced by nonviral delivery of a self-amplifying RNA vaccine expressing HIV type 1 envelope with a cationic nanoemulsion.	J. Infect. Dis.	2015	Bogers WM	NHP	HIV-SAM encoding Env encapsulated in CNE
29263884 (17)	Unmodified mRNA in LNPs constitutes a competitive technology for prophylactic vaccines.	NPJ Vaccines	2017	Lutz J	NHP	LNP-mRNA encoding rabies or influenza antigens
26468547 (18)	Induction of Broad-Based Immunity and Protective Efficacy by Self-amplifying mRNA Vaccines Encoding Influenza Virus Hemagglutinin.	J. Virol.	2016	Brazzoli M	Ferrets, mice	SAM cationic nanoemulsion (CNE) vaccines expressing influenza virus HA
27525409 (19)	Self-Amplifying mRNA Vaccines Expressing Multiple Conserved Influenza Antigens Confer Protection against Homologous and Heterosubtypic Viral Challenge.	PLoS ONE	2016	Magini D	mice	LNP-SAM encoding 2 antigens of influenza virus (NP and M1)
28416600 (20)	Induction of an IFN-Mediated Antiviral Response by a Self-Amplifying RNA Vaccine: Implications for Vaccine Design.	J. Immunol.	2017	Pepini T	mice	LNP-SAM encoding the respiratory syncytial virus (RSV) F protein
31227353 (21)	Co-administration of GM-CSF expressing RNA is a powerful tool to enhance potency of SAM-based vaccines.	Vaccine	2019	Manara C	mice	CNE-SAM encoding the Influenza A virus nucleoprotein (NP)
31290323 (22)	Mannosylation of LNP Results in Improved Potency for Self-Amplifying RNA (SAM) Vaccines.	ACS Infect Dis	2019	Goswami R	mice	LNP-SAM encoding influenza H1N1 antigen HA
26173587 (23)	CD8 T-cell priming upon mRNA vaccination is restricted to bone-marrow-derived antigen-presenting cells and may involve antigen transfer from myocytes.	Immunology	2015	Lazzaro S	mice	LNP-SAM encoding influenza H1N1 antigen HA

The query was performed on December 3^rd^ 2019 searching the following databases: Pubmed, Clinical Trials and USpatent (last update 2019-12-02), EUpatent (last update 2019-11-30).

Scientific literature mining is foundational for the generation of new hypotheses as well as for driving future research and designing new studies. At this point, data and facts of different type and format are gathered and need to be integrated, pressuring data-integration methods to be efficient, in order to explicitly represent the deeply connected big picture the scientist is looking for (24).

## Mechanistic Graphical Modelling

**Figure 2** is a static version of the mechanistic review of the mechanisms of action of RNA vaccines, with a dynamic design made available at the link https://www.cosbi.eu/fx/9839203. Given the known similarities and discrepancies between the mouse and NHP models, we represented separately mouse and NHP data, each of them accessible by clicking on the respective black animal shape in the left upper corner of the model, overlapping the shared information when possible. The immunization process reported in **Figure 2** covers the basics of biology, documented by the references attached to the arrows connecting each element of the model, which have been manually searched while integrating the novelties apported by the mRNA-based vaccine technology, discovered by literature-mining instead.

The mechanistic graphical model starts with either intramuscular or intradermal injection of mRNA, delivered through lipid nanoparticles (LNPs), which showed a more persistent protein expression than systemic intravenous delivery (4). RNA-LNPs enter the cytosol of local neutrophils, monocytes and dendritic cells (DCs), where mRNA expression begins. Immune cells subsequently migrate to the draining lymph node where they orchestrate the T cell and germinal center responses (14). A critical aspect is represented by the way the mRNA vaccine interacts with the sensors in the host’s cells (25). The signaling strength of the exogenous mRNA vector in activating pattern recognition receptors (PPRs), like RIG-I, MDA5 and members of the Toll-like receptors family, is relative to the mRNA species (26–29). This signal is *de facto* a self-adjuvanticity property in the SAM platform that should, at least in principle, be beneficial for the generation of potent immune responses. However, the activation of PPRs is typically associated with the production of type I interferons (IFNs) by plasmacytoid DCs (pDCs) (14) and the induction of the anti-viral state, a condition that has been proposed to severely limit the antibody titers, yet not necessarily impacting the vaccine efficacy (20, 30–32).

The mRNA-based vaccines have shown to most likely leave the injection site through vaccine loaded myeloid DCs (mDCs) making their way to the draining lymph node (dLN) (14), where the concentration of type I IFNs increases, inducing CXCL10, crucial to keep T cells and DCs in proximity, enhancing the chances of T cell activation. After encountering the antigen, both naïve CD4+ and CD8+ become activated and differentiate in mature CD4+ and CD8+ T cells, respectively (14). When CD8+ T cells encounter the antigen and differentiate into short-lived effector cytotoxic T lymphocytes (CTLs), they migrate to the peripheral tissues and to the sites of inflammation (17). In addition, RNA vaccines induce strong Tfh cell responses, which govern the germinal center reactions, including somatic hypermutation, affinity maturation, isotype switching and differentiation of the antigen-specific B cells (15). mRNA vaccines also induce CXCL13, a chemokine responsible for directing B cells efficiently into the follicles (15). From the germinal centers, antigen-specific B cells may differentiate into plasma cells, which home in the bone marrow and continuously secrete antigen-specific antibodies in the blood, or memory B cells, which recirculate in the blood until further antigen encounter (15).

Subsequently, circulating CD4+ and CD8+ T cells may undergo through two possible fates: apoptosis and survival into memory. Indeed, Th1 cells and CTLs may give rise to central memory (CM) CD4+ and CD8+ T cells, respectively. Moreover, depending on the mRNA vaccine doses, both Th1 cells and CTLs have been observed to produce IFNg (15), which is usually associated with strong anti-viral responses, underlying the efficacy of RNA-based therapeutics (15, 17).

## Discussion

mRNA vaccines are emerging as one of the most promising technologies in vaccinology. Several pharmaceutical companies and research institutes are working at the development of new platforms for mRNA-based antigen delivery, trying to identify and characterize those parameters that are required for a safe and protective vaccine response. This, combined with the recent advancements in *omics* technologies, has resulted in the accumulation of a vast amount of data and information. With this work, we built from this unique opportunity and applied text-mining algorithms to screen and analyze scientific literature, with the aim of collecting all available experimentally validated evidence related to the mechanisms of action of mRNA vaccines. We processed and used this information to collect, in a centralized and structured fashion, the various stages of the immune response to mRNA vaccines across different organs and tissues. This knowledge base collection comes in the form of an interactive mechanistic graphical model, which allows to explore the different arms of the immune response to this kind of vaccines.

To maximize the simplicity and interpretability, the model was built using the minimal set of graphical elements, consisting of arrows (representing cells, chemotaxis, transformation or activity) and symbols representing relevant cells and immunoactive peptides. Ideally, a mechanistic graphical model should be readily available to the different stakeholders, should provide some level of interactivity for exploration of the underlying data and be readily upgradable to incorporate new evidences and information. Indeed, an interactive version of the model was made accessible, even remotely, using a web browser, by a proprietary javascript framework. Hosting a copy of the mechanistic graphical model on a remote server ensures future updates to be readily available, avoiding the need of sharing files and risk of misalignments among different versions. Furthermore, all the elements of the mechanistic graphical model can be clicked upon to access the original reference describing that specific evidence. The knowledge gathered during the development of the mechanistic graphical model provides an updated description of the biological phenomena underlying the immune response to mRNA vaccines. The graphical modelling platform can also facilitate the interaction among scientists from different areas, highlight potential gaps in data availability and knowledge and guide the design of new experiments. The natural evolution of this work would be to leverage on the quantitative information acquired during the process (e.g., kinetic parameters, cells concentrations, etc.) to develop a mathematical model describing the immune response, across different biological compartments (e.g. injection site and lymph node) over time (manuscript in preparation). Ideally, this will help highlighting the key elements of an immune response to mRNA vaccines that are responsible for a protective response or, conversely, elements that may lead to suboptimal responses or undesirable effects. Provided an accurate mathematical model is achieved, this could be used to support the experimental design, by allowing to simulate multiple scenarios and predict their outcome. An example could be that of predicting a presumably safe dose range in a dose finding, first time in human clinical study.

As mentioned before, modelling, and prospectively predicting, the behavior of the immune system is a highly challenging task. Consequently, the presented mechanistic graphical model should not be regarded as an endpoint but rather as a milestone within a broader modelling roadmap that we propose as a promising strategy to achieve a better understanding of the human immune system and how it responds to vaccination. The design of future studies for unraveling mRNA mechanism of action will have new pace through the use of literature mining and mathematical modelling, brought together by the power of modern technology.

## Author Contributions

LL, GL, GS and SP computed the results presented in the paper. DT and SG provided technical support in implementing the interactive version of the graphical model. DM, ES and LM conceived the study. ES, GL, LL and LM wrote the manuscript. LL and GL share first authorship. ES and LM share last authorship. All authors contributed to the article and approved the submitted version.

## Funding

This research project was funded by GlaxoSmithKline Biologicals SA.

## Conflict of Interest

GL, HS, DM, and ES were all employees of the GSK group of companies at the time of the study. The “Fondazione The Microsoft Research – University of Trento Centre for Computational and Systems Biology (COSBI)” institute received financial remuneration for conducting the activates described in this study.

The authors declare that this study received funding from GlaxoSmithKline Biologicals SA. The funder had the following involvement in the study: study design, interpretation of data, the writing of this article and the decision to submit it for publication.

The remaining authors declare that the research was conducted in the absence of any commercial or financial relationships that could be construed as a potential conflict of interest.

## Publisher’s Note

All claims expressed in this article are solely those of the authors and do not necessarily represent those of their affiliated organizations, or those of the publisher, the editors and the reviewers. Any product that may be evaluated in this article, or claim that may be made by its manufacturer, is not guaranteed or endorsed by the publisher.
